# Laser Induced Breakdown Spectroscopy for Elemental Analysis in Environmental, Cultural Heritage and Space Applications: A Review of Methods and Results

**DOI:** 10.3390/s100807434

**Published:** 2010-08-09

**Authors:** Rosalba Gaudiuso, Marcella Dell’Aglio, Olga De Pascale, Giorgio S. Senesi, Alessandro De Giacomo

**Affiliations:** 1 Department of Chemistry, University of Bari, *via* Orabona 4, 79126, Bari, Italy; E-Mail: alessandro.degiacomo@ba.imip.cnr.it; 2 IMIP-CNR sec. Bari, *via* Amendola 122/D, 70126, Bari, Italy; E-Mails: marcella.dellaglio@ba.imip.cnr.it (M.D.A.); olga.depascale@ba.imip.cnr.it (O.P.); giorgio.senesi@ba.imip.cnr.it (G.S.S.)

**Keywords:** Laser Induced Breakdown Spectroscopy (LIBS), optical emission, elemental analysis, cultural heritage, soils, meteorites, space exploration

## Abstract

Analytical applications of Laser Induced Breakdown Spectroscopy (LIBS), namely optical emission spectroscopy of laser-induced plasmas, have been constantly growing thanks to its intrinsic conceptual simplicity and versatility. Qualitative and quantitative analysis can be performed by LIBS both by drawing calibration lines and by using calibration-free methods and some of its features, so as fast multi-elemental response, micro-destructiveness, instrumentation portability, have rendered it particularly suitable for analytical applications in the field of environmental science, space exploration and cultural heritage. This review reports and discusses LIBS achievements in these areas and results obtained for soils and aqueous samples, meteorites and terrestrial samples simulating extraterrestrial planets, and cultural heritage samples, including buildings and objects of various kinds.

## 1. Introduction

The very first experiments of laser induced spark emission were performed in the 1960s and involved a laser beam producing a vapour upon irradiation of a solid target and an electrical spark subsequently exciting the ablated plume [1]. These experiments can be considered as early precursors of Laser Induced Breakdown Spectroscopy (LIBS) and since then LIBS has shown its huge potential as an effective technique for fast multi-elemental analysis.

Starting from the 1980s, significant achievements have been achieved in both laser and detector technology, which have made reliable and relatively inexpensive instruments available to research laboratories [2]. This has promoted a huge development of the technique and its adaptation to the qualitative and quantitative analysis of a wide variety of solid samples [3,4]. These include: metal alloys for metallurgy and for jewellery [5–11], cultural heritage materials [12–16], soils, rocks and sediments [17–23], dried and fresh vegetables [24,25], explosives [26]. Gas mixtures and aerosols have also been studied by LIBS [27–30], as well as solutions, for which single- and multi-pulse approaches have been tested [31–34].

What renders the range of applicability of LIBS so wide are primarily its several instrumental advantages, particularly its extremely flexible experimental set-up. Virtually any kind of substance can be analysed by LIBS with no sample pretreatment and no particular sampling procedures needed. Thus, the analysis can be performed in air [35], in vacuum, in fluids [36], and even under extreme conditions such as high temperature and pressure environments. This enables to design compact and portable LIBS instruments, fit for *in situ* [37,38] measurements and for applications such as space exploration [39–43], as well as potentially adaptable to on flight analysis of asteroids and to stand-off analyses [44]. Besides, LIBS provides a fast response and is thus suitable for on-line process control [45], has a good sensitivity (generally of the order of ppm) and induces a limited and localized damage on the sample surface, being the removed amount on the order of tens or hundreds of nanograms.

LIBS can be easily coupled with other laser-induced spectroscopic techniques, primarily Raman spectroscopy and Laser-Induced Fluorescence (LIF) (see for example [46,47]). These unique and attractive features qualify LIBS as a technique highly competitive with other largely applied spectroscopic methods for elemental analysis, such as Inductively Coupled Plasma-Optical Emission Spectroscopy (ICP-OES) and Inductively Coupled Plasma-Mass Spectrometry (ICP-MS). In particular, it is often the technique of choice, in some cases coupled with other techniques such as Raman spectroscopy, for geochemical and cultural heritage applications, due to their particular demands for non-destructiveness, speed of analysis and possibility of performing *in situ* and remote analysis.

On the other hand, the main drawbacks of LIBS include: its strict dependence on the fulfilment of equilibrium conditions in the plasma; the possible saturation of the signal for high concentration elements due to self-absorption effects, even for high-energy lines, that makes their quantification difficult or impossible with LIBS; the onset of matrix effects when analysing samples of very different kinds or very inhomogeneous specimens; the large number of complex physical-chemical phenomena involved in the processes of ablation and plasma formation, evolution and interaction with the background ambient.

In the present paper, the Laser Induced Plasmas (LIP) fundamentals and LIBS analytical methods are outlined, and the recent achievements obtained by our research group in the field of elemental analysis for cultural heritage, environmental and space applications are reviewed and inserted in the frame of the main questions the LIBS community has being addressing in the 2000s [48–50].

## 2. Basic Principles of LIP and LIBS

When a short laser pulse (with typical duration from ns to fs), is focused on a portion of matter, a significant amount of energy is transferred to the latter, which can result in the formation of a plasma of the irradiated material, a phenomenon usually referred to as *breakdown* at the material surface. The breakdown can occur only if the pulse irradiance exceeds a threshold value which depends on the state of aggregation of the material [51]; an irradiance value of ∼1 GW/cm^2^ is generally considered as an appropriate reference value to yield a high-temperature and high-electron density plasma from virtually any kind of irradiated solid targets. Once ablated, the material expands at supersonic velocity (in vacuum the initial expansion velocity is ∼10^6^ cm/s) in a direction orthogonal to the target (approximately, expansion along the radial component can be neglected in the early stages after ablation). LIP is not a stationary system and typical values for temperature and electron density of LIPs in air range, respectively, between 20,000 K and 10^19^ cm^−3^ in the early times after its formation, and 5,000–6,000 K and 10^17^ cm^−3^ at the late stages of its evolution.

Several phenomena can contribute, at different extents, to produce the breakdown and yield the plasma, depending on the features of both the laser (wavelength, pulse duration, irradiance) and the irradiated material (state of aggregation, physicochemical characteristics). During its finite lifetime (∼10 μs in air), the produced plasma plume emits radiation that can be detected and used to retrieve qualitative and quantitative information about the elemental composition of the irradiated target, provided that the ablation is stoichiometric, namely, that the composition of the luminous plasma is the same as that of the target. Due to its transient nature, LIP manifests departures from the thermodynamic equilibrium due to the escape of radiation, that is, to radiative dissipation of part of the internal energy of the system. Anyway, such energy loss is negligible with respect to that exchanged by processes between material particles, which are therefore locally and instantaneously in equilibrium and are established in a shorter time than that required for the plasma to expand. Such condition is referred to as Local Thermodynamic Equilibrium (LTE) [52] and its occurrence is the underlying premise for the use of LIBS as an analytical technique, as it will be better clarified in the following section.

**Figure 1.**
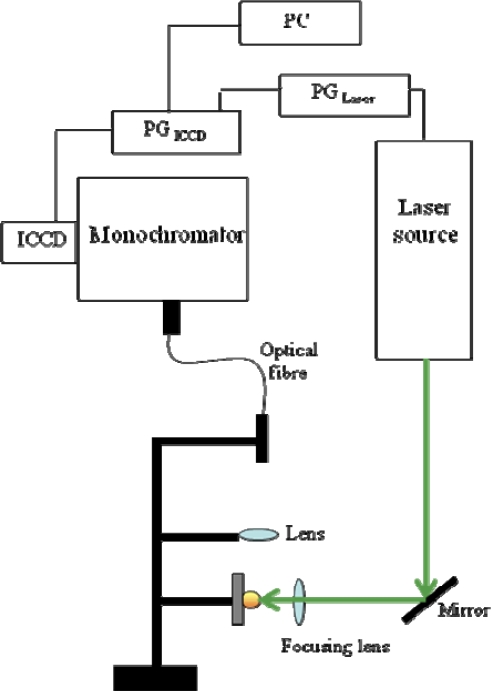
Block diagram of a typical experimental set-up for Single Pulse LIBS experiments. The acronym PG stands for Pulse Generator.

An example of a typical experimental set-up for LIBS experiments is provided in Figure 1, where the instrumentation generally employed in our laboratory is sketched as a block diagram. It mainly consists of a laser source (in our case, a nanosecond Q-switched solid-state laser, Nd:YAG, with first harmonic at 1,064 nm and second harmonic at 532 nm), a system for the radiation detection that comprises a monochromator coupled with an Intensified Charge Coupled Device (ICCD), and a pulse generator to synchronize the plasma production and the emission spectra acquisition. The emitted light can be collected through an optical fibre, as depicted in Figure 1, or directly focused on the monochromator entrance slit. Acquisition parameters, including number of spectra accumulations and averages, starting delay time and gate width of ICCD aperture with respect to laser pulse, can be set *via* software by means of a computer interfaced with the spectrograph. It is important to note that in LIBS experiments the solid targets are usually mounted on a rotating sample holder, in order to prevent the sample from being deeply dug in the point where the laser beam is focused. Moreover, using this solution, spectra can be acquired from several points on the target surface and all contribute to the overall signal, thus accounting for possible surface inhomogeneity that may affect the accuracy of the analytical determination.

Another common configuration is the Double Pulse LIBS (DP-LIBS), which involves the use of two time-delayed laser pulses to enhance the emission spectra intensity and improve the limits of detection by using either a collinear or an orthogonal geometry. In the first case the two collinear pulses follow the same optical path and reach the target surface perpendicularly with a time delay that can be set according to the background environment [53]. In the second case only one pulse ablates the target, while the other is directed parallel to its surface, with two possible temporal schemes. If the parallel pulse is shot before the ablative pulse, it can produce a preparative pre-spark in air [54]. If it is shot after, it can re-heat the ablated material [55]. In any of the mentioned double-pulse configurations the two lasers can have the same or different pulse widths or wavelengths.

A collinear double pulse configuration can also be used to induce the breakdown of liquid samples, which proceeds with plasma formation mechanisms that are completely different from those occurring in solids. In particular, a single laser pulse is not appropriate for the analysis of bulk liquid samples, because most laser energy is spent in the interaction with the surrounding liquid bulk, which rapidly quenches the plasma. Therefore, a double-pulse approach is necessary for the analysis of submerged targets and liquid bulks, most typically water solutions. The plasma produced by the first pulse rapidly extinguishes, producing a cavitation bubble within the aqueous bulk; the second pulse induces a plasma inside the bubble which evolves similarly to LIPs in gaseous background and can be therefore useful for analytical purposes.

A detailed discussion about the laser-water interaction and the plasma generation under water is beyond the scope of this paper, but the interested reader can refer to previous works of ours and of other groups for a comprehensive treatment [56–58]. In the following sections, short mentions will be given to some applications of analytical LIBS to liquids (aqueous matrices) and submerged solids.

## 3. Elemental Analysis by LIBS

The use of LIBS for elemental analysis relies on some fundamental assumptions that must be verified, especially when semi-quantitative and quantitative analysis of samples is pursued. First of all the conditions of optically thin plasma and LTE are always the underlying premises for laser induced plasma studies by emission spectroscopy, both for fundamental studies and for analytical applications.

The emission intensity *I_ul_* of a peak of frequency *v_ul_* is related to the number density of excited emitters, *N_u_* by:

(1) $$I_{\mathrm{ul}}=\frac{1}{4\pi }N_{u}A_{\mathrm{ul}}{\mathrm{hv}}_{\mathrm{ul}}G$$

Where *A_ul_* is the spontaneous emission coefficient of the given transition, *h* is the Planck constant and *G* is an instrumental factor depending on the experimental set-up used [59]. This relation is valid only when the considered *ul* transition is optically thin, that is, not suffering from self-absorption, a condition that can severely affect low-energy and resonance lines, particularly for high-concentration elements.

Several methods can be found in the literature [60–62] to evaluate and correct self-absorption, and a good practice in analytical LIBS is to select accurately the emission lines to be used for analysis. With this regard, some line selection criteria have been proposed by our group [21] and are briefly recalled here:

- All spectroscopic lines involving the ground state should be excluded, because they are the most severely affected by self-absorption phenomena, which alter the spectral line profiles and thus hinder the quantitative determination of major elements. For these elements, lines corresponding to transitions with lower energy level below 6,000 cm^−1^ should also be possibly excluded.
- All transitions with spontaneous emission rate lower than 2 × 10^6^ s^−1^ should not be considered for quantitative analysis because the corresponding emission times might be comparable with the time associated to the plasma variations [63].
- All emission lines with high relative intensity (generally >3,000) should be considered with care. In fact, when the emission of low intensity transitions is under the detection limit, e.g., close to the plasma border, these lines are still detectable, therefore they can be useful for the detection of trace elements but they have high probability to overestimate the population, especially for elements at high concentration.

Two further aspects to be considered in the use of analytical LIBS are stoichiometric ablation and plasma homogeneity. All the ablation-based analytical techniques, such as LA-ICP-OES or LA-ICP-MS, as well as LIBS itself, are affected by the stoichiometry of material removal from the target, which may be altered by matrix effects, such as preferential ablation, or fractionation, of some species. The fractionation issue has been addressed in a number of papers [2,64–67] due to the occurrence of non-linear calibration graphs of emission intensity as a function of analyte concentration for reference samples. Fractionation effects are often present in LIBS analysis, in dependence on the matrix under study. For some of them, for example copper-based alloys, the effect can be minimised to reach an acceptable uncertainty of concentration determination by choosing appropriate laser parameters, such as fluence, wavelength, pulse duration, and employing calibration techniques to improve the analytical performance, though they may never be truly overcome [68].

On the other hand, establishing plasma homogeneity is a more complex issue. As a matter of fact, spatial and temporal gradients of temperature and both electron and heavy species number densities arise in the plasma, due to its fast dynamics and its interaction with the background environment. The peripheral zones of the plasma are the most involved in phenomena such as recombination processes (between ions and electrons within the plasma and between species from the surrounding ambient and plasma species) and confinement by the shock wave contact wall. Consequently, differences may arise between plasma parameters in external zones and those in plasma core. Anyway, for diagnostic or analytical purposes, only the portion coming from the plasma core is usually employed. For this sampled portion, the homogeneity assumption holds, as demonstrated by several authors with spatially-resolved LIBS spectra (see [67] and references therein). The validity of this assumption can be further ensured by means of appropriate experimental approaches, such as choice of temporal and spatial regions suitable for spectra detection, and methods of data treatment for spatial resolution, such as the Abel inversion [59].

As for the LTE condition, it is common practice in the LIBS literature to consider that the elementary balances between material particles are in equilibrium, while the light-matter balance is not, if the Boltzmann distribution (see next section) and the McWhirter criterion hold. This criterion states the existence of a critical value for electron density *N_e_*, that is:

(2) $$N_{e}({\mathrm{cm}}^{-3})>1.6\times 10^{12}T^{\frac{1}{2}}(K){\left(\Delta E_{\mathrm{nm}}\right)}^{3}(\mathrm{eV})$$

where T is the plasma temperature and Δ*E_nm_* is the maximum energy gap between two adjacent transition levels n and m. Though a detailed discussion about the LTE condition in LIPs is beyond the scope of the present paper, it is useful to mention here that the McWhirter criterion is actually only a necessary condition for LTE, but its use has been widely documented for all analytical purposes in LIBS literature (see for example [69] and references therein). A further requirement is that the acquisition time parameters are accurately chosen to make sure that the Saha equilibrium of ionization/recombination reactions is established in a time shorter than that associated to the change of plasma parameters. For details about the assessment of LTE in LIPs, the interested reader can refer to [52,70,71].

## 4. LIBS Approaches to Quantitative Analysis

Two traditional approaches are possible for quantitative analysis by LIBS, a calibration line approach and a calibration free procedure. The first consists in drawing a calibration line with a set of standards of the same kind and of similar composition to that of the unknown sample. As shown in Equation (1), the intensity of a given emission line is proportional to the number density of emitters, which, in turn, is proportional to the concentration of the emitter (namely, the analyte) in the irradiated sample. Therefore, the emission intensity is linearly correlated to the concentration of a given species in the sample, I_X_ = K[X]. In order to minimize the matrix effects, which could affect the calibration graph linearity, the emission intensity is usually normalized to that of the most abundant element in the sample, which is used as an internal standard. Another possible approach for the calibration line is to normalize the analyte signal to the intensity of the spectral background signal [17].

Furthermore, multivariate calibration techniques can be used for quantitative analysis by LIBS, such as Partial Least Squares regression (PLS) and Artificial Neural Networks (ANN), which have been drawing increasing attention from the LIBS community over the past few years (see for example [72,73]). Such advanced calibration techniques can reduce the complexity of spectra, enabling to extract the valuable information when the standard calibration curve method may fail. Moreover, chemometric methods such as Principal Components Analysis (PCA) are often coupled to calibration techniques in order to observe data clusters and discriminate outliers, prior to performing the actual quantitative determination [38,74–76].

An alternative to calibration approaches is the so-called Calibration Free (CF) or Self-Calibration method, which is based on the LTE assumption and was first proposed by Ciucci *et al*. [77]. In LTE conditions, the number density of emitters is given by the Boltzmann distribution:

(3) $$N_{u}=\frac{g_{u}N_{0}}{Z(T)}\exp\left(-\frac{E_{u}}{\mathrm{kT}}\right)$$

where g_u_ is the statistical weight of the excited level of energy E_u_ from which the emission occurs, N_0_ is the total number density of the given species, Z(T) is the partition function at the excitation temperature T. By substituting N_u_, as derived from the Boltzmann distribution, in Equation (1) and putting in a logarithmic form, the equation of a line is obtained:

(4) $$\ln\frac{I_{\mathrm{ul}}4\pi }{A_{\mathrm{ul}}{\mathrm{hv}}_{\mathrm{ul}}g_{u}}=\ln\frac{N_{0}G}{Z}-\frac{E_{u}}{\mathrm{kT}}$$

where an instrumental factor appears, G, which cannot be evaluated unless a radiometric calibration is performed.

This method, commonly referred to as the Boltzmann plot method, enables the determination of the excitation temperature from the line slope and N_0_ from the intercept. N_0_ is proportional to the species weight percentage of species in the sample, which can be retrieved as described in [77] through a normalization procedure:

(5) $$\sum_{i}{w\%}_{i}=x\sum_{i}N_{0,i}{\mathrm{AW}}_{i}=1$$

where AW_i_ is the atomic weight of the species i and the normalization constant x also contains the instrumental factor G. Once x has been determined, each percentage is given by

(6) $${w\%}_{i}=x\;N_{0,i}{\mathrm{AW}}_{i}$$

In order for the CF approach to be used for quantitative measurements, the above mentioned assumptions (stoichiometric ablation, LTE condition, plasma homogeneity, optical thin plasma) must be strictly fulfilled.

The CF algorithm represents the only possible approach when the preparation of matrix-matched standards is unfeasible or difficult (e.g., in extreme environments, such as nuclear reactors) and in attaining automated analytical procedures. However, the accuracy of CF quantitative measurements depends more critically on the necessary assumptions for analytical LIBS and may lead to only semi-quantitative results when their fulfilment is unsatisfactory [67,68]. The results that will be presented in the following sections have been achieved mostly by CF-LIBS or by calibration methods, either with the standard calibration line approach or with multivariate techniques.

## 5. LIBS in Cultural Heritage Science

The most appealing features of LIBS applications in the field of cultural heritage analysis are mainly the following: its micro-destructiveness, with ablated sample portions on the order of fractions of micrograms and induced damage virtually invisible to the naked eye; its potential for fast multi-elemental analysis and capability of simultaneous detection of major and trace elements; its equipment, easily compactable into portable instruments for *in situ* analyses of piece of arts that cannot be removed from museums or excavation sites, or of historical buildings and wall paintings.

LIBS appears the most suitable technique for dating and rapid classification purposes of metal objects that are among the most abundant archaeological findings, including tools, weapons and utensils, as well as jewellery and statues. Copper-based alloys were the most used in the ancient times and several LIBS analytical and fundamental studies have been dedicated to ancient bronzes, partly because of their suffering from fractionation effects [2,64–66] mainly due to the significantly different melting points of their main constituents. Fantoni *et al*. [2,68] performed a modelling study aimed at clarifying the fractionation effects that could be expected upon ablation of quaternary bronzes (Cu, Zn, Sn, Pb) with different lasers, namely, single ns-pulse with different (IR to UV) wavelengths, double ns-pulse, single fs-pulse. By comparing the concentrations in the plasma as results of simulations of the different ablation mechanisms with certified concentration values, these authors identified the possible deviations from the stoichiometric ablation and provided a method to optimize the experimental conditions.

A different approach, based on the experimental comparison between ns- and fs-ablation of copper-based alloys, was proposed by our group in [5], in order to establish if significant differences might arise between the analytical performances of laser pulses of different duration. In [5] a variant of the CF method was developed which was based on the assumption of partial LTE (pLTE) in the energy range covered by the observed emission lines in the fs and ns cases. The number densities of plasma species were determined by normalization of the line emission intensity to that of the continuum background, which was assumed to follow a Planck-like distribution. With this method, the concentrations of minor elements (Sn, Pb, Ni) in three bronze standards were calculated, while Cu concentration was not determined due to the significant self-absorption of Cu lines, and its weight percentage was just considered as the complementary species for the determined ones. The obtained results, summarized in Table 1, showed that a significant agreement exists between fs- and ns-CF-LIBS, in spite of the known marked differences in the ablation mechanisms of solid targets by ultra-short and short pulses [79–81]. Data in Table 1 show that a good agreement exists between the ns- and fs-LIBS analytical results, as well as between them and the certified values for the concentrations of some elements (Sn, Pb, Ni). It appears therefore reasonable to consider that fractionation was negligible in both cases and that the residual fractionation took place in a comparable degree for both pulse durations.

Once established the suitability of ns-LIBS for quantitative analysis of bronze samples, in a subsequent work [12], we analyzed by a ns Nd:YAG laser five VII b.C. century objects made of copper-based alloys, namely, a belt (A), a basin (B), a brooch or *fibula* (C), a piece of armour fragment (D) and a pendant (E), which were excavated from an archaeological site in Southern Italy (Minervino Murge, Apulia). First of all, a depth profiling of Pb and Sn was performed on a piece of scarce archaeological interest (a basin fragment, which was not included in the above selection), in order to estimate any possible discrepancy in their concentrations between the sample surface and bulk. Indeed, it is well known that the external layers of ancient objects strongly differ from the original material as a result of long-time corrosion, so that both morphology and composition can be altered significantly. In the case of bronze artefacts, this corrosion process is often referred to as “bronze disease” which, due to the object interaction with its burial environment (soil, water or sea water), causes it to be covered by a layer (patina) containing mainly Cu II salts and, in some cases, also significant amounts of Sn compounds [82].

**Figure 2.**
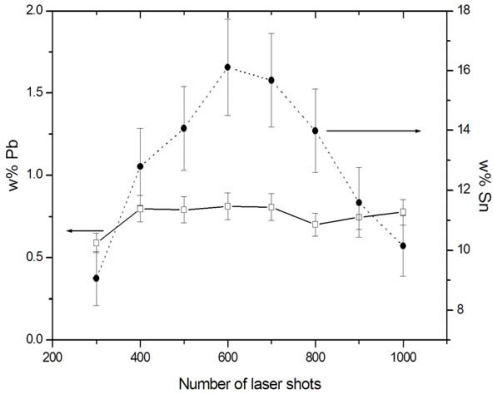
Sn and Pb weight percentages (w%) in a fragment of a bronze basin as a function of the number of laser shots focused on the sample. Each spectrum resulted from 100 accumulations, with laser energy 1.5 mJ and focused laser spot of diameter 200 μm. The sample was drilled through from side to side, displaying a significant depletion of Sn in the external layers.

The possibility of analysing multi-layered samples and performing depth profiling is virtually unique to LIBS, among the various techniques used for elemental analysis of archaeological objects. This is because the laser beam focused in a given spot removes successive layers thus different spectra can be observed as a consequence of the compositional changes. In the case of metal and stone handiworks, the incoming laser beam is able to locally remove the altered layers, so that in the irradiated spot the bulk materials, with composition close to the original one, can be analyzed. In particular, in the case of bronze artworks it is important to perform a depth profile analysis to establish how many laser shots are needed to reach the bulk sample, where the composition holds a constant value and can be considered reasonably close to the original one. The Sn and Pb concentrations of the basin fragment are plotted in Figure 2 as a function of the number of laser shots reaching the sample. In this experiment, 600 shots were necessary to remove the patina and reach the bulk material. The end point of the corrosion layer was also made visually evident by the appearance of the typical red-brownish bronze colour. It is interesting to note that, while Pb maintains an almost constant concentration across the sample section, Sn shows a marked depletion in the outer layers, possibly due to the formation of Sn compounds and their subsequent solubilisation in the presence of moisture in the burial soil.

For the quantitative analysis of the above-mentioned unknown samples, a classical calibration line approach was preferred, due to the mentioned difficulty of determining Cu concentration with a CF method. In this approach, Cu was used as the internal standard to draw calibration lines for the quantitative analysis of Sn, Pb, Zn, Fe, Ni, Sb and Si.

A useful element for dating studies is As, which was not present in the analyzed samples. Dating studies of this kind, aimed at distinguishing between arsenical bronzes, typical of Early Bronze Age, and tin bronzes, which were used from the Middle Bronze Age on, have been performed by Fortes *et al*. [14]. These authors examined a set of 37 specimens from Southern Spain and, based on their As content, sorted them in Bronze Age and Iron Age objects.

**Figure 3.**
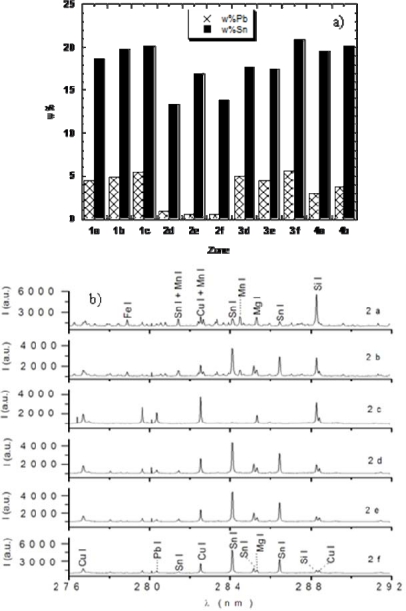
**(a)** Bar charts of Sn and Pb weight percentages at increasing sampling depth in the four nailed parts constituting the bronze belt samples A; **(b)** spectra of the zone repaired from the external layers 2(a,b,c) to the internal ones (2(d,e,f), used for the bar chart in Figure 3 (a)). The spectra from the external layers show typical features from the soil elements (Si, Mg) and were not used for the quantitative analysis, while increasing the sampling depth peaks of bronze components (Cu, Pb, Sn) appear. It is interesting to note that the spectrum 2(c) lacks the Sn peaks, which indicates some inhomogeneity in the alloy composition, and was therefore not used for the analysis.

In the case of the Italian bronzes analyzed in our laboratory in collaboration with a team of archaeologists, we drew some conclusions about the possible use of the various hand works, on the basis of their Sn content. For example, the piece D was found to have a high Sn content (17 ± 1 wt%), which suggested that it was a part of a piece of armour, probably a helm, because adding large amounts Sn to the alloy hardened the material and prevented it from being easily damaged by blows. On the other hand, unusually high concentrations of Sn and Pb were found in specimens C and E, lying outside the range covered by the available standards. Thus, for these samples only lower limits could be obtained for concentrations (Sn > 39 ± 3 wt%, Pb > 27 ± 2 wt% for the pendant; Sn > 28 ± 2 wt%, Pb 4.3 ± 0.3 wt% for the fibula), which were sufficient to suggest that they must be pieces of jewellery. Indeed, they are not made of common bronzes, but of slightly different copper-based alloys respectively named as *bell metal* and *high leaded tin bronze*, which, due to their high Sn percentage, were difficult to tool and used for precious objects. The B sample was a piece of basin, with a well conserved rim and showing typical tin bronze concentrations (Sn 7.6 ± 0.5, Pb 0.17 ± 0.03) which allowed an easy working of the piece, as required to obtain every day use objects of various shapes. The most interesting piece of the set was sample A, a belt composed of four pieces nailed together. For each piece, a depth profiling was performed and Sn and Pb were determined with increasing sampling depth. The obtained results were quite surprising and are reported in Figure 3(a,b): three of the four nailed pieces had typical tin bronze concentrations, while one had a significantly lower Pb content. This suggested that the fourth piece was probably added to the hand work in a later time, likely by a different craftsman, to repair the belt after it got damaged. Nails were also analyzed and they resulted composed of Fe and C, but these components were not quantified.

As an example of the depth profiling procedure, the six spectra of the added piece (Zone 2) are reported in Figure 3. Apparently, the spectra of the external layers are contaminated by high amounts of soil elements such as Si, Mg (see Figure 3(b), Zone 2(a)), while those of the internal layers, used for the quantitative analysis, only display the typical spectral features of bronze elements (see Figure 3(b), Zone 2(f)). These results clearly highlight the significant advantages of using LIBS for this kind of analysis. First of all, no piece was sacrificed, but only a negligible damage was caused, directly on the sample surfaces, with a spot diameter around 180 μm. Moreover, depth profiles were easily performed, which enabled to discriminate the corrosion layer and to make sure that the analyzed sample portion was representative of the original material, at the same time removing the insignificant or extraneous ones.

The spectra of removed layers can also provide useful information to conservators, helping them to identify the causes of object alteration and to plan tailored restoration and/or cleaning actions [13]. Since laser cleaning of a wide variety of artworks has been strongly developing in the last decade, LIBS has become an effective method for on-line monitoring of the cleaning process itself. During contaminants removal from the object surface, the emitted spectra change due to change of elemental composition of the ablated layer. This change can be used to understand the causes of contamination and also to avoid undesired over-cleaning of the object itself. Laser cleaning, with the aid of on-line LIBS monitoring, has been employed to remove extraneous coatings of various kinds, including dark encrustations from marble, terracotta, stone and glass artworks due to exposure to air pollutants [16,83–85], protective and conservative coatings from canvas paintings and synthetic mimicking materials [86–90], corroded layers from metal objects [91,92], dirt (dust and pen and pencil traces) from historical paper documents [93,94], pencil and pen marks on alabaster and marble statues in a stand-off configuration [95], and patinas on surfaces of historical buildings [96].

Besides being a powerful technique to control the removal of layers due to aging and pollution of artworks, LIBS is among the few techniques able to provide multi-elemental depth-profiling of intrinsically multi-layered samples, such as ceramics, paintings and frescoes, resulting at the same time only micro-destructive. This potential has been exploited, often coupled with Raman spectroscopy that provides complementary molecular information for dating and provenance studies [97,98] and for pigment identification in a number of different painted artworks, including ceramics [99,100], icons and miniatures [101,102], painted plasters [103], polychromes on wood [104], artistic prints [105], wall paintings of historical buildings [106–107] and historical parchment [15].

In conclusion of this section, our studies on submerged bronze targets will be briefly mentioned [32]. These studies were conducted in the frame of a project aimed at verifying the feasibility of LIBS analysis for drowned artefacts, such as sunken ships and their content, directly *in situ* (namely, under seawater). The possibility of obtaining some information about the findings would be of primary importance prior to planning recovery activities and taking decisions about whether the object should be moved or not. In our work, bronze standards immersed in seawater were analyzed in a home-made cuvette with two collinear laser pulses focused through the liquid bulk. The quantitative analysis was obtained with the calibration line method and gave satisfactory results, as compared to similar ones performed in air. The calibration lines for Sn and Pb under water had higher slope and correlation coefficient than those obtained in air, which indicated higher sensitivity and better accuracy. This result suggested that a better thermalization through electron impact took place under water, due to the strong confinement experienced by plasma species inside the bubble, which, together with a longer-lasting LTE condition and a more homogeneous plasma, led to a better analytical performance [32].

## 6. LIBS in Environmental Science

The interest of LIBS in the broad field of environmental science lies mainly in its versatility and potential for *in situ* measurements with a very limited sample pretreatment or none at all. The discussion in this section will be focused on the analysis of soil in its various aspects. Soil is the environmental compartment most affected by heavy metal contamination, being constantly exposed to metal emissions from many anthropogenic sources [108], and thus potentially subject to long-term modifications, with consequences on the equilibrium of ecosystems. Other kinds of samples will also be included in the discussion, such as rocks and sediments, due to the wide variety of specimens studied by various authors especially during *in situ* and remote analyses. For a further discussion specifically focused on the LIBS analysis of rocks and minerals, the interested reader may refer to [109] and references therein. Finally, a short mention will be given in this section to the analysis of aqueous samples, namely seawater and wastewater from industrial plants, due to the strong need for rapid *in situ* chemical sensors in such field and the potential of LIBS to fulfil such need.

### 6.1. LIBS Analysis of Soils

Soil analysis is one of the most traditional applications of LIBS. Compared to LIBS, the techniques most typically used for this purpose (such as Atomic Absorption Spectrometry (AAS), X-Ray Fluorescence (XRF), ICP-MS, ICP-OES) and the chemical methods specific for the various analytes, generally regulated by national or international protocols (see for example [110,111]), usually provide better LODs but are time-consuming, need consistent sample manipulation prior to analysis, and require specific soil sampling procedures and accurate specimen selection. Further, and especially for the detection of contaminants in soils after accidental or systematic spill of toxic waste, the use of a real-time, *in situ* analytical technique such as LIBS would allow the assessment of their degree and kind of contamination, followed by immediate application of tailored remediation strategies, in order to avoid irreversible damages to environment and to human and animal health.

The detection and measurement of levels of toxic metals in soils are among the most important concerns of environmental science, due to the important implications that excessive depletion or enrichment of trace elements in soils can have on the health of living species [108,112]. This is by far the most studied environmental issue as far as LIBS is concerned. In the last decade, many research groups have been developing experimental strategies for this purpose, their common long-term aim being the optimization of reliable LIBS procedures for the assessment of soil degree of contamination and the development of fast remediation strategies. In this context, LIBS research proceeds along two parallel directions: on one hand, the laboratory optimization of LIBS experiments, in order to overcome the many problems of direct analysis of an untreated, exceptionally complex matrix such as soil; on the other hand, the development of high-performance instruments for on field and stand-off investigations.

#### 6.1.1. Laboratory LIBS Analysis of Soils

**Figure 4.**
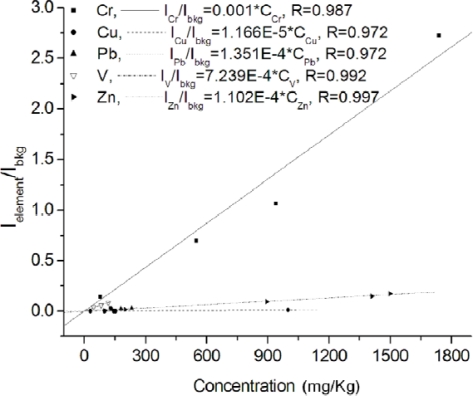
Calibration lines of Cr, Cu, Pb, V, Zn in samples of polluted soils and sewage sludge collected in various Italian regions.

Since the 1990s many papers have already appeared in the LIBS literature concerning the laboratory analysis of soil samples. Research in this domain is still very active and is mainly aimed at refining the technique and evaluating its capabilities in comparison with other well established techniques. Capitelli *et al*. [20] determined the concentration of Cr, Cu, Fe, Mn, Ni, Pb and Zn in a heterogeneous set of soil and sludge samples of various origin and nature and compared the LIBS performance to that of ICP-OES. These two techniques, which can be considered similar in that they are both based on the detection of light emitted by a high-temperature and high-density plasma, were also compared in a work of our group [17] where the contents of Pb, Zn, Cu, V, Cr were determined in five soil and sludge samples, finely grained and pressed in pellets with no further pretreatment. Calibration lines (reported in Figure 4) were drawn on the basis of ICP-OES measurements. For these lines the emission intensity of analytes was normalized to that of the spectral background, because no suitable internal standard was found in the analyzed set of samples. The good linearity of calibration lines was a clear confirmation of the feasibility of the adopted method and showed that limited matrix effect took place.

In order to compare the LIBS performance with that of ICP-OES, the following procedure was followed for each element. One sample, chosen from the central part of the concentration range covered, was excluded from the calibration curve of the considered element and treated as an unknown sample. A new calibration line was then drawn without the excluded sample, and the concentration of the element was determined consequently. The results of such comparisons are reported in Table 2 and show a good agreement between the two techniques.

**Figure 5.**
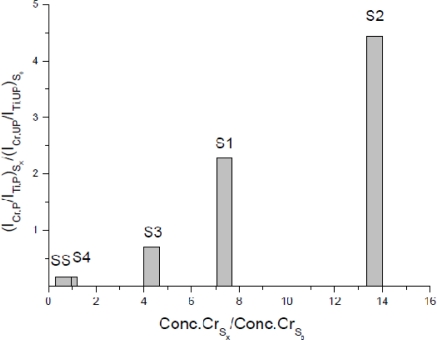
Anthropogenic index calculated with Cr emission intensities (line at 520.45 nm) for samples of polluted soils (S1, S2, S3, S4) and sewage sludge (SS). S1 and S2 are two loam soils from Apulia, Southern Italy; S3 a clay loam from Lombardy, Northern Italy; S4 a sandy clay loam from Marche, Central Italy.

In the same paper, we defined an anthropogenic index (A.I.) to rapidly evaluate the degree of heavy metals pollution of soils, in analogy to other enrichment factors proposed in the agrochemical literature [113]. The A.I. was defined as the ratio between the concentration of an element in a polluted soil normalized to that of a reference element, and the corresponding value in an unpolluted soil of the same kind. Taking into account that for a given analyte a direct proportionality exists between line emission intensity and sample concentration (see Equation 1), a modified A.I., relying on LIBS intensity instead of concentration, was defined as:

(7) IM,PITi,PIM,UPITi,UP=A.I.

where the subscript M indicates the given analyte and the subscripts P, UP stand for polluted and un-polluted soil. The modified A.I. was calculated for Cr, using Ti as the reference element, in five of the examined samples, using the sixth as the reference soil. The bar chart in Figure 5 shows the achieved results plotted as functions of the ratio of Cr concentration in the analyzed soil over that in the reference soil. This alternative approach for the A.I. determination is very fast and particularly suitable in view of *in situ* applications, such as fast pre-evaluation of severely heavy metals-polluted soils. In conclusion, this approach allows the estimation of the degree of pollution of a soil with no need of concentration calculations, but only on the basis of the emission intensities.

Several further works have been published on this subject. For example, Hussain *et al*. [114] determined the concentration of Cr, Ca, Fe, Mg, Cu, Na, Ni, K and Ba in soil samples collected from a coastal area surrounding the Khursania site of Saudi Arabia along the Persian Gulf, which was involved in a large oil spill during the Gulf War. Another highly toxic contaminant, Cd, was the object of the investigation by Santos *et al*. [115] who evaluated its concentration in standard soil and sediment samples. Bousquet *et al*. [116] determined Cr in soils collected from industrial areas or prepared in the laboratory by comparing the results obtained with different emission lines and by LIBS and ICP-OES.

The allowed concentration of some heavy metals in unpolluted soil is very low (see for example the limits established by the Italian Ministry of Environment for Hg and Cd, respectively 1 mg/kg and 2 mg/kg in public parks and residential areas, which rise to 5 mg/kg and 15 mg/kg in industrial and commercial areas [117]). Therefore, some alternative approaches to the classical LIBS have been proposed to improve the sensitivity of the technique, including the use of double pulse irradiation [118,119].

Besides the assessment of the soil degree of pollution by heavy metals, LIBS has also been proposed for other soil-related studies, including soil classification by means of chemometric methods [116,120]; monitoring of the phytoremediation process of heavy metals-polluted soils [121–123]; establishing soil quality, both in terms of micronutrients content [124] and to aid the evaluation of soil organic matter (SOM) and fertility by means of total C and N quantitative determination [19,125–127].

#### 6.1.2. *In situ* LIBS Analysis of Soils

As regards the development of LIBS portable systems for direct *in situ* analysis of soils, so far most efforts have been dedicated to preliminary laboratory testing and calibration of mobile instruments and to addressing the important matrix problems that can be expected during such analyses. For example, Ferreira *et al*. [128] have calibrated a portable instrument through laboratory measurements, using a chemometric method based on the use of Artificial Neural Network (ANN) for Cu determination in a heterogeneous set of Brazilian soils, though poor correlation was found between Cu intensity and concentration.

Bousquet *et al*. [38] designed and built a prototype of a mobile instrument, equipped with a Nd:YAG ns-laser with the fundamental harmonic and tested it in laboratory, addressing the most serious issue affecting the feasibility of *in situ* LIBS analysis of soils, namely, the influence of soil moisture on the analytical performance. They attempted two possible approaches to solve this problem. The first consisted in irradiating the samples with long IR pulses, in order to evaporate the excess water prior to focusing the ablative pulse. The second was the classical approach of pressing the samples into pellets to squeeze out water. Of these two strategies, only the latter provided satisfactory results. In all the experiments reviewed so far, matrix effects in quantitative analysis were limited by using either univariate calibration strategies, *i.e.*, the usual calibration line approach, or multivariate ones, e.g., ANN and PLS.

Differently, Corsi *et al*. [129] used the CF algorithm (described in Section 4 of this paper), to overcome the calibration problem and also to reduce markedly the time analysis to 1 to 5 minutes, according to the complexity of the produced LIBS spectrum. Their experiments were carried out in the laboratory, in order to test the response of a mobile instrument, equipped so as to analyze either soil pellets or directly the “unsampled ground”. Moreover, they performed a comparative study between the plasma produced by a single laser pulse and by a sequence of two pulses. This study confirmed the DP-LIBS potential for lowering the LODs and increasing the emission intensity and the technique sensitivity for the considered elements. However, the main drawback of using the CF approach for soil analysis is that some of the main soil elements are also contained in the surrounding gas (*i.e.*, air). As a consequence, their weight percentage in the ablated samples cannot be easily quantified, because the spectral signals from air and from the target can be distinguished only if high spatial resolution is applied in the spectra acquisition (see for example [130]).

Results of measurements actually performed on site have already been published. Wainner *et al*. [131] compared the sensitivity of a portable spectrometer and a laboratory instrument for the specific detection of Pb in soils and paint. In this experiment collected samples were analyzed in the laboratory, after doping them with variable amounts of Pb-containing powders or solutions. Then, the portable system was tested on areas of documented Pb contamination, namely, the grounds around a military installation in California used until the late 1950s for the demilitarization by burning of small arms ammunition and Pb-containing paints on the walls of an old military hospital in Colorado. In both cases, analyses performed on site were only qualitative, but allowed the authors to validate the portable system for the detection of Pb concentrations down to 0.01% in weight.

A different kind of study was carried out by Barbini *et al*. [22] by using a portable LIBS instrument mounted on board the R/V Italica ship during the XVI Antarctic campaign (2000–2001). Sediment slices of 2 cm diameter sampled from the 30 cm diameter and 50 cm height cylindrical sections extracted from the sea bed were oven-dried and then qualitatively analyzed to identify the presence and distribution of various elements in the different sediment horizons, with the aim of locating the most interesting sites with a fast on line method.

The quantitative analysis of two selected pressed sediments was performed with a method proposed by the same group in [18] and based on the evaluation of self absorption in the core zone of the plasma while neglecting that in the external zones. The obtained results were compared with certified values, resulting in a satisfactory agreement except for Al, which resulted severely underestimated by LIBS.

Recently, quantitative analysis of Pb in road sediments has been reported by Cuñat *et al*. [23] by means of a man-portable LIBS instrument, consisting of a backpack and a probe unit of overall weight of approximately 5 kg and a size of 45 cm × 27 cm × 15 cm. An on-field campaign was performed on soil samples in the Cerrado de Calderon tunnel in Malaga, Spain, to evaluate the Pb released by the road traffic. Depth-profiling of Pb and Ti concentrations, normalized to that of Ca, were performed on the tunnel walls every 2 m along the tunnel length. The comparison of results obtained by LIBS quantitative analysis with those of AAS laboratory analysis of samples collected in the gallery, indicated the existence of a good correlation between the two techniques and a satisfactory LOD which resulted lower than those demanded by most environmental organizations.

#### 6.1.3. Stand-off LIBS Analysis of Soils

Stand-off LIBS applications have also been described for soils analysis. For example, López-Moreno *et al*. [132] investigated samples from a coastal site subjected to a high industrial activity and therefore to potentially significant heavy metal contamination. These samples, including rocks, soils, trees leaves and bark, stone and wall fragments from the factory or from surrounding buildings, were qualitatively and quantitatively analyzed in laboratory, at a stand-off distance of 12 m. The effects of the sample conditions (*i.e.*, salinity, moisture) and characteristics (*i.e.*, matrix composition, orientation with respect to the incoming laser, distance from the contamination source) were investigated. Further, depth-profiling of rocks and stone samples clearly revealed a surface contamination by Cr whose emission concentration was unexpectedly high on the samples surface and decreased until eventually disappearing in the bulk of the unpolluted sample.

In a later paper [133], the same authors described the design, construction and assessment of a field-deployable instrument for open path remote LIBS, which was optimized for acquisitions in a hundreds of metres range. The authors investigated which critical parameters should be taken into account in stand-off experiments by using acquisition tests on Ti as a reference sample and at increasing distances. An equation was retrieved for the LIBS signal dependence on the acquisition range (distance from the irradiated target), laser wavelength, beam divergence and dimensions of the receiving optical element, which are factors more critical in a stand-off configuration than in laboratory experiments. Further, the analyte emission was compared to that of air components, in order to evaluate any interference due to possible breakdown along the laser beam path.

### 6.2. LIBS Analysis of Aqueous Samples

From the environmental point of view, what renders LIBS in liquid environment particularly interesting with respect to well-established techniques for liquids analysis such as ICP-OES [134] is the possibility of planning on-site and remote applications, dedicated essentially to monitor the concentration of metals in wastewaters from industrial plants and in seawater. As briefly discussed in Section 2, focusing a single laser pulse through a liquid bulk does not usually provide suitable analytical signal, but low emission spectra mainly dominated by a continuum background radiation, where only few low-energy lines can be observed [56,57].

Though some works report results about the detection of various elements by focusing a laser in bulk aqueous samples (Koch *et al*. for Cr [135], De Giacomo *et al*. for Al, Na, Ca and Li in various water solutions [31]), over the past several years different Single-Pulse (SP) configurations have been tried to overcome the intrinsic drawbacks of the traditional configuration. For example, Hussain *et al*. in [136] determined the concentrations of Ca, Mg, P, Si, Fe, Na and K in wastewater samples from dairy products plants. The laser beam was focused on the liquid surface contained in a suitably designed cell so as to avoid splashing on the optical components, and high energy laser pulses (100 mJ) were used to enhance the emission intensity. This approach provided accurate analytical results that were in good agreement with those obtained with ICP-OES of the same samples.

Rai *et al*. [137] used a liquid jet configuration to detect Cr in wastewater from electroplating industries. High energy pulses (120 mJ) were focused just below the surface of a solution stream from a Teflon nozzle, so to produce a plasma at its front surface. Good agreement was found between the LIBS and AAS results for the only analyzed unknown sample, provided that a suitable calibration line could be drawn prior to the LIBS experiment.

The liquid stream approach was also adopted by Cheung *et al*. [138,139], who used an ArF excimer laser to ablate the sample. They obtained good results for the analysis of water solutions, with LODs on the order of ppb, thanks to the VUV-laser wavelength, which provided a better signal-to-noise ratio and a shorter-lived continuum emission than those usually found with non-VUV-LIBS.

However, for bulk liquids and submerged targets the best improvements can be obtained by using a Double-Pulse (DP) approach. The cavitation bubble generated by the extinction of the first pulse offers a favourable microenvironment for chemical analysis and represents the only possible choice if liquid bulk analysis is pursued, as in the case of *in situ* analysis of seawater. In one of our works, previously cited for the analysis of submerged bronze samples, a laboratory analysis of tap water was also performed which showed significant improvements for both qualitative and quantitative analysis made possible by the DP approach [32].

An interesting application, proposed by Michel *et al*. [36,140], is the use of LIBS as a chemical sensor at high liquid pressures simulating oceanic depths, aimed at *in situ* identification of hydrothermal vents. Clearly, the effects of quenching and spectral interference by continuum radiation are even more severe at high pressures (up to hundreds of bars), which also limit the lifetime of the bubble itself and the temporal windows when the analysis can be pursued. However, it is important to underline that LIBS in the cavitation bubble appears as the only possible choice for the analysis of hydrothermal vents, whose emitted fluids can be observed only *in situ*, because irreversible changes would occur if they were moved from underwater to a common laboratory [140]. The effect of some key experimental parameters such as delay between the laser pulses, background pressure, pulse energies and acquisition gate time were studied and optimized for the detection of five critical elements (Na, Ca, Mn, Mg, K).

## 7. LIBS in Space Science

All the mentioned advantages of LIBS as an analytical technique are particularly attractive for its use in space applications. LIBS feasibility as an *in situ* and remote technique renders it particularly appropriate for space exploration purposes, also because LIBS instruments can be integrated in rovers for planetary surface analysis and easily coupled with Raman spectrometers for simultaneous multi-elemental and molecular information. In particular, the next NASA and ESA missions to Mars (respectively Mars Science Laboratory, scheduled for launch in Fall 2011 [141] and ExoMars, scheduled for launch in 2016 [142]) will include also a compact spectrometer for stand-off LIBS analysis on the respective rovers. Moreover, LIBS has a specific strong advantage in the similarity between LIPs and the ablation plasmas produced by falling meteoroids while crossing the Earth atmosphere [143–145]. Laser ablation techniques can be therefore a useful resource to simulate such spectra and contribute to asteroids and meteorites recognition. In particular, the mass of flying bodies can be determined by analysis of their luminous ablation plasmas and their elemental composition, which in turn allows predicting their trajectory and the effect of their impact [146,147]. Further, the optimization of LIBS experiments in laboratory conditions controlled is expected to promote future development of on-flight techniques for the direct analysis of flying meteoroids.

The elemental analysis of meteorites, which is employed for their classification, can be performed on a geological basis (petrological and mineralogical classifications) and/or on a chemical one [148], depending on the kind of meteorite. With respect to established techniques usually employed for this aim, including INAA (Instrumental Neutron Activation Analysis), PIXE (Proton-Induced X-ray Emission), ICP-MS and XRF, LIBS presents the obvious advantages of not requiring any analytical chamber or radioactive sources and of being adaptable also to extreme conditions and out of the laboratory, with no limitations of the atomic weight of the detectable elements. Therefore, research in the field of LIBS spatial applications proceeds dealing with both the elemental analysis of actual space objects and the development of experimental strategies and prototypes for planet exploration, mainly performed on terrestrial samples similar to Martian and Lunar ones.

Thompson *et al*. [43,149,150] have analyzed two Martian meteorites, Zagami and Dar al Gani 476, in a stand-off mode at distances of up to 8.3 m, aiming to test laboratory systems as similar as possible to the ChemCam instrument built for the NASA Mars Science Laboratory and to develop calibration strategies for improving the method reliability and reduce the matrix effects on the analytical performance. Calibration lines have also been drawn using terrestrial basalts to test the remote-LIBS capability of distinguishing between the two different Martian meteorites.

In a work of ours [21], a CF method was employed to analyze a set of four known meteorite samples of different classes, namely an L6 chondrite (Sahara 98222), a Lunar meteorite (Dhofar 461), a Martian meteorite (Dhofar 019) and an iron meteorite (Sikhote Alin). The weight percentages of the main elements were determined, while the trace elements were detected only qualitatively, and the LIBS results were compared with those in the literature, obtained by INAA and ICP-MS. Due to the difficulty in preparing a satisfactory set of matrix-matched standards for the analysis of meteorites of different classes and, above all, to the impossibility of applying calibration procedures in the case of completely unknown samples, we opted for the CF-LIBS approach. The obtained results were in good agreement with the weight percentages reported in the literature for most elements, although the CF accuracy was still not very good (around 15%).

**Figure 6.**
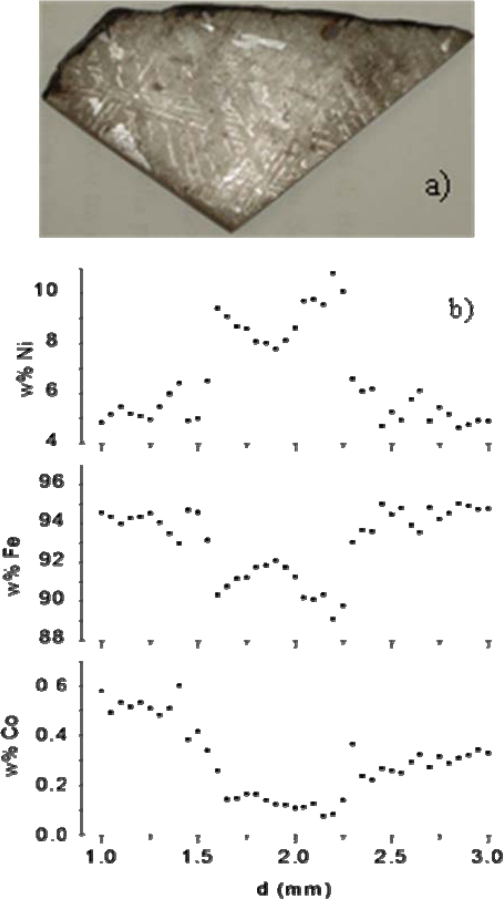
**(a)** Slab of Toluca iron meteorite (medium octahedrite, class IAB) displaying on its surface the peculiar Widmanstätten pattern; **(b)** weight percentage profile of Ni, Fe and Co across one lamella of the Widmanstätten structure of Toluca.

Successively, we studied in detail two further iron meteorites, Campo del Cielo and Toluca [151] and for the very first time in LIBS literature, we obtained concentration profiles of the main elements, Fe and Ni, and of the most abundant trace element, Co, along the Widmanstätten structure, visible on the Toluca meteorite analyzed slab (see Figure 6(a)). This is a pattern of alternating taenite and kamacite *lamellae*, peculiar to iron meteorites of the Toluca class (medium octahedrites) and its detection, coupled with theoretical models, is an important method for the study of the parent body, the mechanism of formation and the age of meteorites [148]. The space resolution achieved by the used LIBS experimental set-up (laser spot diameter 25 μm) was not comparable with that of techniques that use electron or ion microprobes, but enabled anyway the clear identification of seven lamellae and the determination of the main elements concentrations across the Widmanstätten pattern. The weight percentages of Ni, Fe and Co across one lamella are reported in Figure 6(b) as an example. It is noteworthy that the spatial resolution could be easily improved by using a micro-LIBS set-up, which consists of a microscope through which the laser beam can be focused, thus obtaining micrometric spots. The good agreement found with a simple cooling rate theoretical model [152] demonstrated the potential competitiveness of LIBS with the classical techniques for meteorites analysis. In [151], we also determined the oxygen content of the Martian meteorite Dhofar 019 by using a CF method and irradiating the sample in reduced pressure Ar atmosphere.

So far, most work of the LIBS community in the field of space applications has been dedicated to the analysis, mainly at short stand-off distance, of rocks and materials simulating surface and atmosphere of extraterrestrial planets. The purpose of these studies is the optimization of prototypes and methods for forthcoming planetary exploration missions, which must fulfil stringent requirements for the dimensions of instrumentation. To this aim many European and US groups are working, in the frame of the above mentioned ESA and NASA missions to Mars.

Colao *et al*. [153] performed CF-LIBS analysis of Martian rocks analogues (volcanic and sedimentary rock collected near the Etna Volcano in Italy and on Chilean Andes) in a special analytical chamber, in a rarefied gaseous environment (CO_2_:N_2_:O_2_ = 95:4:1, total pressure adjusted between 6 and 9 Torr) simulating composition and low pressure of the Martian atmosphere. Prior to Martian analogues investigation, these authors validated the CF method for the analysis of complex matrices by using several soil and rock standards, and found a substantial agreement between LIBS values and certified values. The most important deviations were observed for easily ionizable elements, especially Ca.

Similar preparatory studies have been carried out by several authors. Sallé *et al*. [154] tested a stand-off system for qualitative analysis of a set of metallic alloys and basalt samples in the range 2–12 m, and detected the major components up to 12 m and the trace elements up to 8 m. A different configuration, at a distance of 3 m, was used for quantitative analysis, particularly of Cl and S, two elements often difficult to detect by LIBS due to their high excitation energies (≥ 10 eV) but extremely important for the study of Martian geology. For this purpose, samples containing Cl and S including KCl, NaCl, pure S, FeS_2_, PbS, CuS, were analyzed, in order to produce a spectral database and calibration lines. Experiments were conducted in simulated Martian atmosphere, a feature that was found to increase the signal to background ratio of Cl and S emission lines and improve their detection limit.

Radziemski *et al*. [155] investigated the feasibility of acquiring spectra in the VUV region (particularly 100–200 nm) for the analysis of Cl, S and other important elements, such as As, Br, C, P, of difficult detection in the regions normally covered by most LIBS spectrometers (visible-near IR). Also in this case, soils, minerals and metal samples were analyzed in simulated Martian atmosphere and used to draw calibration lines. A good linearity was obtained for calibration lines and the precision was comparable with that of usual LIBS measurements at longer wavelengths, but the authors pointed out an intrinsic drawback. As CO_2_ absorbs in the region 130–160 nm, the feasibility of such method at long stand-off distances in Martian atmosphere may be limited, being however suitable on asteroids and on the Moon.

Further experiments simulating Mars and Moon atmospheres (respectively, 7 Torr CO_2_ and near vacuum conditions, 50 m Torr in air) were performed by Sallé *et al.* [42], who studied the influence of the ambient pressure on calibration curves prepared for elements commonly observed in terrestrial soils (e.g., Al, Ba, Ca, Cr, Fe, K, Li, Mg, Mn, Na, Ni, Si, Sr, Ti) by using certified soil and clay pellets as standard samples. In a later work [156], these authors compared four different analytical methods, *i.e.*, multi-matrices calibration curves, external normalization with correction for total concentrations, internal normalization by a major element and CF approach, in order to select the one with the best performance in overcoming matrix effects. The analyzed samples were terrestrial rocks similar to Martian ones, *i.e.*, mafic volcanic rocks, gabbro and limestone, which were irradiated in CO_2_ at 9 m bar, at an intermediate stand-off distance (3 m). The best accuracy was obtained using the external normalization method, which consists in the determination of a matrix factor accounting for the difference in the number of ablated atoms of a given species between the standard and the unknown samples. This approach, as the CF one, is useful in case no reference sample is available but may not be very accurate and requires the detection of all elements. When this is not possible, *i.e.*, in the case only a limited portion of the spectrum can be acquired, the internal standardization method is considered a valid alternative, as it can be applied only to the detected analyte.

In addition to the atmosphere composition, another factor which has been taken into account by some groups to simulate Mars conditions is the presence of ice and the moisture degree of Martian rocks. The presence of water, as already discussed in the section dedicated to *in situ* analysis of soils, can affect the laser-target coupling and consequently the LIBS signal intensity. On the other hand, it can give a significant contribution to verify current or past presence of life on the Red Planet.

Arp *et al*. [157] simulated the analysis of polar layer deposits at Mars poles by acquiring at short and intermediate stand-off distance LIBS spectra at Martian atmospheric pressure of several kinds of samples, including pure water ice, mixtures of ice and synthetic silicate certified materials mimicking soils and mixtures of ice and powdered certified rocks of various types. The effects on LIBS spectra of adding increasing amounts of ice to soil samples was studied and the penetration properties of the laser pulse through frozen samples were determined. Furthermore, the LODs evaluated at short distance and in a stand-off mode for dry soils and ice/soil mixtures yielded comparable results.

Lazic *et al*. in [40] applied cooling and heating cycles to terrestrial soil and rock samples in a simulated Martian atmosphere, in order to investigate the effect of the presence of ice and water in the samples pores on the emission spectra, as a function of temperature in the range from 25 °C to −60 °C. The preparation of samples, that is, their surface roughness and pore dimensions, was found to significantly affect the emission spectra. In particular, these effects were ascribed to the phase transitions undergone by the supercooled water present in the sample pores and, in particular, to the decrease in the laser-sample coupling due to discontinuities in the physical properties of the coexisting liquid water and ice phases. In a later work [41] these authors further refined their investigations by performing measurements at pressures below and above the triple point of water, and found in the latter case a clear anti-correlation between H emission intensity (used as a marker of water vaporization) and the sum of the emission intensities of the main elements constituting the rock samples.

Finally, at least a short mention should be given here to the exploration of other extraterrestrial planets. Although Mars is the most studied, due to its resemblance to Earth and its relatively mild surface conditions, other planets have received some attention, such as for example Venus. Its exploration by lander missions is challenging due to its extremely harsh conditions (CO_2_ atmosphere, 90 atm pressure and 735 K surface temperature). The high pressure and temperature would limit the lifetime of any surface probes to no longer than few hours, therefore fast *in situ* analytical techniques such as LIBS would be required.

A preliminary study of LIBS feasibility for Venus missions was reported by Arp *et al*. [39], who acquired LIBS spectra of basalt rock samples in a high pressure chamber filled with pure CO_2_ or CO_2_/N_2_ mixtures at high pressure. They investigated the effect of experimental features such as the temporal evolution of emission signals and the dependence on the pulse energy.

More recently, papers about coupled LIBS-Raman analysis of Venus-analogue rocks in Venus-like atmosphere have appeared [159], which demonstrate that some useful information can be retrieved, though LIBS spectra are complicated by the appearance of emission lines that are not typically observable under terrestrial conditions and are caused by the increased number of collisions between plasma species and the surrounding supercritical CO_2_. A primary concern for the use of emission spectroscopy in Venus conditions is represented by the quenching of the LIBS signal, due to pressure effects which decrease the plasma lifetime, the transmission properties of CO_2_ and the continuum background interference in Raman spectra. Specific approaches and experimental strategies are required to address such issues, which were first investigated by Lambert *et al*. [160], in view of future development of an on-flight instrument for LIBS-Raman analysis.

## 8. Conclusions

The present paper has reviewed LIBS applications to qualitative and quantitative elemental analysis in the broad fields of environmental science, space exploration and cultural heritage. LIBS is a multi-elemental technique consisting in the production of a luminous transient plasma by focusing an intense laser pulse (of ns or less duration for typical analytical applications) on a target and in the subsequent detection of the plasma emission spectra. The main approaches to quantitative analysis by LIBS have been discussed and examples have been provided of analytical results obtained using either the classical calibration line approach (with some variants according to the calibration strategy adopted) or the Calibration Free approach. The LIBS capability of providing the elemental composition of virtually any kind of samples with no sample pretreatment and its potential for *in situ* and remote analysis, even in harsh environments, together with its micro-destructiveness and comparatively low cost, renders this technique particularly suitable for the analysis of a wide range of samples.

This review has focused on the analysis of various kinds of artworks, with a special emphasis on archaeological findings made of copper-based alloys, soils and meteorites and space objects. Examples of investigations performed with portable instruments for *in situ* LIBS measurements and of stand-off analyses, especially in the environmental and space exploration fields, have also been briefly presented and discussed, together with the main problems associated to the use of these variants of the standard laboratory technique. A short mention has been finally given to the double-pulse LIBS technique, used for the analysis of submerged solid targets or of bulk liquids, together with results about its application in the cultural heritage and environmental fields.

## Figures and Tables

**Table 1.** Comparison between the CF-LIBS results for three bronze standards upon irradiation by ns- and fs-lasers. The experimental uncertainty was estimated to range between 10–15%.

**Sample**	**Certified w%**	**fs-CF-LIBS w%**	**ns-CF-LIBS w%**
**B4**	Cu 84.00	Cu −	Cu −
	Sn 11.05	Sn 9.32	Sn 9.70
	Pb 2.50	Pb 3.99	Pb 4.11
	Ni 0.57	Ni 0.81	Ni 0.31
**B21**	Cu 83.05	Cu −	Cu −
	Sn 5.13	Sn 5.73	Sn 4.02
	Pb 3.79	Pb 3.30	Pb 4.46
	Ni 1.21	Ni 1.10	Ni 1.65
**B22**	Cu 82.75	Cu −	Cu −
	Sn 3.85	Sn 4.15	Sn 3.25
	Pb 6.12	Pb 6.76	Pb 6.38
	Ni 2.56	Ni 1.62	Ni 2.90

**Table 2.** Comparison between concentrations of heavy metals in soil samples determined by ICP-OES and by LIBS (calibration line method).

**Element**	**Unknown sample**	**ICP-OES concentration (mg kg^−1^)**	**LIBS concentration (mg kg^−1^)**
Cr	S3	550 ± 82	698 ± 144
Cu	S4	100 ± 15	88 ± 21
Zn	S2	897 ± 135	846 ± 37
Pb	S1	180 ± 27	168 ± 18
V	S1	84 ± 12	80 ± 7
